# The fungal natural product azaphilone-9 binds to HuR and inhibits HuR-RNA interaction *in vitro*

**DOI:** 10.1371/journal.pone.0175471

**Published:** 2017-04-17

**Authors:** Kawaljit Kaur, Xiaoqing Wu, James K. Fields, David K. Johnson, Lan Lan, Miranda Pratt, Amber D. Somoza, Clay C. C. Wang, John Karanicolas, Berl R. Oakley, Liang Xu, Roberto N. De Guzman

**Affiliations:** 1 Department of Molecular Biosciences, University of Kansas, Lawrence, Kansas, United States of America; 2 Molecular Graphics and Modeling Laboratory and the Computational Chemical Biology Core, University of Kansas, Lawrence, Kansas, United States of America; 3 Department of Chemistry, University of Southern California, Los Angeles, California, United States of America; 4 Department of Pharmacology and Pharmaceutical Sciences, School of Pharmacy, University of Southern California, Los Angeles, California United States of America; 5 Center for Computational Biology, University of Kansas, Lawrence, Kansas, United States of America; BSRC 'Alexander FLEMING', GREECE

## Abstract

The RNA-binding protein Hu antigen R (HuR) binds to AU-rich elements (ARE) in the 3’-untranslated region (UTR) of target mRNAs. The HuR-ARE interactions stabilize many oncogenic mRNAs that play important roles in tumorigenesis. Thus, small molecules that interfere with the HuR-ARE interaction could potentially inhibit cancer cell growth and progression. Using a fluorescence polarization (FP) competition assay, we identified the compound azaphilone-9 (AZA-9) derived from the fungal natural product asperbenzaldehyde, binds to HuR and inhibits HuR-ARE interaction (IC_50_ ~1.2 μM). Results from surface plasmon resonance (SPR) verified the direct binding of AZA-9 to HuR. NMR methods mapped the RNA-binding interface of HuR and identified the involvement of critical RNA-binding residues in binding of AZA-9. Computational docking was then used to propose a likely binding site for AZA-9 in the RNA-binding cleft of HuR. Our results show that AZA-9 blocks key RNA-binding residues of HuR and disrupts HuR-RNA interactions *in vitro*. This knowledge is needed in developing more potent AZA-9 derivatives that could lead to new cancer therapy.

## Introduction

The limited lifetime and subsequent decay of messenger RNA (mRNA) is an important mechanism for posttranscriptional regulation of gene expression. In mammalian cells, mRNA decay is dependent on both *cis* elements located in the RNA and *trans* acting regulatory factors such as RNA-binding proteins. AU-rich elements (ARE) in 3’-untranslated region (UTR) of mRNAs are common *cis* elements that promote rapid degradation of mRNAs [1, 2]. Specific RNA-binding proteins can bind to AREs and either accelerate decay or protect mRNA from degradation [1–4].

The RNA-binding protein Hu antigen R (HuR), a ubiquitous member of the ELAV/Hu protein family, binds and stabilizes ARE-containing mRNAs that encode oncoproteins, cytokines, growth factors and transcription factors [3–7]. HuR is a multi-domain protein containing three RNA-recognition motifs, RRM1, RRM2, and RRM3, with each RRM comprising of about 80 amino acids. High affinity binding of HuR to ARE of mRNA is accomplished via its two tandem N-terminal RRM, RRM1 and RRM2 that are separated by a 7-residue inter-domain linker [8]. The third RRM of HuR, RRM3, along with the basic hinge region that connects RRM2 with RRM3 mediate cooperative assembly of HuR oligomers on RNA [9]. Although HuR is predominantly nuclear, the protein rapidly translocates to the cytoplasm in response to stimuli mediated by a nucleo-cytoplasmic shuttling sequence located in the hinge region [10]. Further, HuR is phosphorylated, ubiquitinylated, and methylated; and these posttranslational modifications of HuR affect its RNA-binding, subcellular localization, and stability (reviewed in [11]).

HuR is overexpressed in a wide variety of cancers, including colon, ovarian, brain, breast, cervical, and pancreas [7, 12–14]. HuR promotes tumorigenesis by binding to cancer-associated ARE-containing mRNAs that encode proteins implicated in tumor cell proliferation, cell survival, angiogenesis, invasion, and metastasis [7, 15–17]. HuR binds and stabilizes the AREs of the oncogene Musashi1 (Msi1) and anti-apoptotic proteins, Bcl2 and XIAP, thereby up-regulating their expression and activating the Wnt/Notch signaling pathway and inhibiting apoptosis [15, 18, 19]. Disrupting HuR-ARE interaction is thus an attractive strategy in developing new cancer therapeutics [16, 20–22]; and small molecule inhibitors of HuR have been reported [16, 23–25].

We have previously reported screening of ~6000 small molecule compounds for HuR inhibitors using a fluorescence polarization assay [22]. To expand the known chemical space of HuR inhibitors [16, 22–25], we report here that azaphilones inhibit HuR-RNA interaction. Azaphilones are derived from the fungal natural product asperbenzaldeyde [26, 27]). We characterized the HuR-binding of the most potent azaphilone derivative, azaphilone-9 (AZA-9), by fluorescence polarization (FP), surface plasmon resonance (SPR), nuclear magnetic resonance (NMR), and computational modeling. AZA-9 disrupts HuR-RNA interaction by competitive binding in the RNA-binding cleft of HuR.

## Materials and methods

### Protein expression and purification

The protein expression and purification of full length HuR (326 residues) and HuR RRM1/2 (residues 18–186) have been described [22]. For NMR studies, in addition to ^15^N-labeling, we also used ILV-labeling, where the Isoleucine Cδ1 and the geminal Leucine Cδ and Valine Cγ methyl groups are ^13^C-labeled by growing *E*. *coli* in M9 minimal media supplied with ^13^C alpha keto acids. His_6_-tagged HuR RRM1/2 simultaneously labeled with ^15^N and ILV was prepared by expression in *E*. *coli* BL21 (DE3) grown in 1 liter of M9 minimal media supplemented with 1 g of ^15^N-ammonium chloride and 3 g of glucose at 37°C. At OD_600_ ~0.4, the growth medium was supplied with 60 mg of 2-ketobutyric acid-4-^13^C (Sigma #571342) to label the ^13^Cδ1 methyl group of isoleucine and 100 mg of 2-keto-3-(methyl-^13^C)-butyric acid-4-^13^C (Sigma #571334) to label the two leucine ^13^Cδ and the two valine ^13^Cγ methyl groups [28]. Approximately 1 hour later (at OD_600_ of ~0.8), the culture was induced with 0.7 mM isopropyl-β-D-thiogalactopyranoside (IPTG), and cell growth was continued overnight in a 15°C shaker incubator to a final OD_600_ ~2.5. Cells were harvested by centrifugation (2,400 × *g*, 10 min), resuspended in binding buffer (30 mL; 500 mM NaCl, 20 mM Tris-HCl pH 8.0, 5 mM imidazole), and lysed by sonication in the presence of 0.1 mM phenylmethanesulfonylfluoride (PMSF). Cellular debris was removed by centrifugation (13,900 × *g*, 10 min), and 600 μL of 5% (v/v) polyethyleneimine was added to the supernatant to precipitate the nucleic acids. Following centrifugation (13,900 × *g*, 10 min), the supernatant was loaded into a 5 mL Ni^2+^ column, washed with binding buffer and the His_6_-tagged HuR RRM1/2 was eluted with elution buffer (500 mM NaCl, 20 mM Tris-HCl pH 8.0, 250 mM imidazole). The HuR RRM1/2 construct used herein retained an N-terminal His_6_-tag. Purified protein was dialyzed in buffer (100 mM NaCl, 10 mM NaPO_4_ pH 6.8) and concentrated using Amicon Ultra 3K centrifugal filter (Millipore). Protein concentration was determined by A_280_.

### ILV assignment

Mutagenesis was used to assign the ILV resonances by introducing conservative mutations (I→L, L→I, and V→I) in HuR RRM1/2. The QuikChange kit (Stratagene) was used to introduce site-directed mutants and mutations were verified by DNA sequencing. Proteins obtained from cell growth in 200 mL of M9 minimal media supplied with the appropriate ^13^C-alpha keto acid was enough to obtain 2D ^1^H,^13^C HSQC to assign the ILV peaks of the 12 isoleucine and L39, L61, V66, and L138 residues of HuR RRM1/2.

### Chemicals and reagents

Synthetic RNA oligos were from Dharmacon. For FP assay, fluorescein-tagged ARE^Msi1^ oligo derived from the 3’-UTR of Musashi RNA-binding protein 1 (Msi1) with the sequence 5’-GCUUUUAUUUAUUUUG-3’ and 11-mer ARE^c-fos^ RNA oligo (5’-AUUUUUAUUUU-3’) identical to the c-fos RNA sequence used in the crystal structure of HuR-RNA complex [8] were used. For NMR studies, unmodified 11-mer ARE^c-fos^ RNA oligo [8] was used. Prior to the addition of RNA to the protein, the RNA was heated at 95°C for 5 min followed by immediate cooling on ice for 5 min. The azaphilone derivatives used herein were obtained by semisynthetic diversification of asperbenzaldehyde, which was purified from a strain of *Aspergillus nidulans* that was engineered to overproduce this compound as described elsewhere [26, 27]. Compounds were dissolved in dimethyl sulfoxide (DMSO) to form 10 mM stock solutions; for NMR studies, deuterated dimethyl sulfoxide (d_6_-DMSO) was used.

### Biochemical assays

FP competition assay for screening and hit validation were carried out as reported previously [22]. Briefly, compounds at increasing doses were added to plate wells prior to the addition of pre-formed protein-ARE^Msi1^ or protein-ARE^c-fos^ complex. To form HuR-ARE complex, 10 nM full length HuR and 2 nM Msi1 oligo or c-fos oligo were used. To form HuR RRM1/2- ARE^c-fos^ complex, 50 nM HuR RRM1/2 and 2 nM c-fos oligo were used. Measurements were taken using a BioTek Synergy H4 hybrid plate reader (Biotek, Winooski, VT) after incubating for 2 hours at room temperature. IC_50_, the drug concentration causing 50% inhibition, was calculated by sigmoid fitting of the dose response curve using GraphPad Prism 5.0. Percent inhibition was calculated by comparing to the DMSO (0% inhibition) and labeled free RNA only (100% inhibition) controls.

Surface plasmon resonance (SPR) datasets were acquired using a BIACORE 3000 (GE Healthcare) at 20°C as described [22]. Briefly, HuR was immobilized into a CM5 chip by amine-coupling chemistry and AZA-9 dissolved in buffer (20 mM HEPES pH 7.4, 150 mM NaCl, 3 mM EDTA, 0.05% p20 (v/v), 5% DMSO (v/v)) was injected into the flow cell at a flow rate of 60 μL/min. The mixtures complexes were allowed to associate for 400 sec and dissociate for 160 sec. SPR sensorgrams were generated using Scrubber2 (BioLogic Software, Australia).

### NMR spectroscopy

Proteins for NMR were dissolved in NMR buffer (100 mM NaCl, 10 mM NaPO_4_ pH 6.8, 10% D_2_O). ^15^N NMR data were acquired using Bruker Avance 800 MHz spectrometer with a TCI cryoprobe. ILV ^13^C NMR data were acquired using on a Bruker Avance III 600 MHz spectrometer. NMR data were acquired at 25°C, processed using NMRPipe [29] and analyzed using NMRView [30]. For ^15^N and ILV chemical shift mapping, 80 μM ^15^N/ILV-labeled HuR RRM1/2 was titrated with unlabeled ARE^c-fos^ RNA at increasing molar ratios of 1:0, 1:0.5, 1:1, and 1:1.7. AZA-9 was titrated at 1:1 and 1:2 molar ratios into 50 μM ^15^N/ ILV-labeled HuR RRM1/2 in NMR buffer with 10% d_6_-DMSO. Higher protein concentrations or higher molar ratios of AZA-9 resulted in sample precipitation. The ^15^N titrations were monitored by acquiring 2D ^1^H-^15^N TROSY spectra, and the ILV titrations were monitored by acquiring 2D ^1^H-^13^C HSQC spectra. The published backbone amide assignments of HuR RRM1/2 (BMRB entry 26628) [20] and the ILV assignments made herein were used in the NMR analysis.

### Computational modeling

A docked model of how AZA-9 may bind to HuR RRM1/2 was built starting from the crystal structure of HuR RRM1/2 in complex with RNA (PDB 4ED5) [8]. FRED (version 3.0.1) from OpenEye Software was used for molecular docking [31]. For each of the biological units present in PDB 4ED5, the RNA was removed and a receptor was built using APOPDB2RECEPTOR (OpenEye Scientific Software, Santa Fe, NM). Conformers of AZA-9 were generated with OMEGA (version 2.5.1.4) [32] using the default parameters and docked into each of the prepared receptors using FRED at the “Standard” docking resolution. Full-atom minimization of the model with the best score based on the Chemgauss4 scoring function was then carried out using ROSETTA [33] using the Talaris2014 scoring function with the pwSHO solvation model [34].

## Results

### Protein expression and purification of HuR and HuR RRM1/2

Full length HuR (326 residues) and a shorter HuR construct consisting only of the two tandem RRM1 and RRM2 (HuR RRM1/2; residues 18–186) were overexpressed in *E*. *coli* BL21 (DE3) and purified under native conditions by Ni^2+^-affinity chromatography (S1 Fig). Typically, ~28 mg of pure unlabeled or ^15^N/ILV-labeled recombinant HuR RRM1/2 protein was obtained per liter of LB or M9 minimal medium. The HuR RRM1/2 was soluble in 100 mM NaCl, 10 mM sodium phosphate pH 6.8 buffer at a concentration of ~6 mg/mL (~0.27 mM protein concentration), beyond which, it precipitated at higher concentrations.

### Identification of AZA-9 as an inhibitor of HuR-RNA interaction

**Fig 1:**
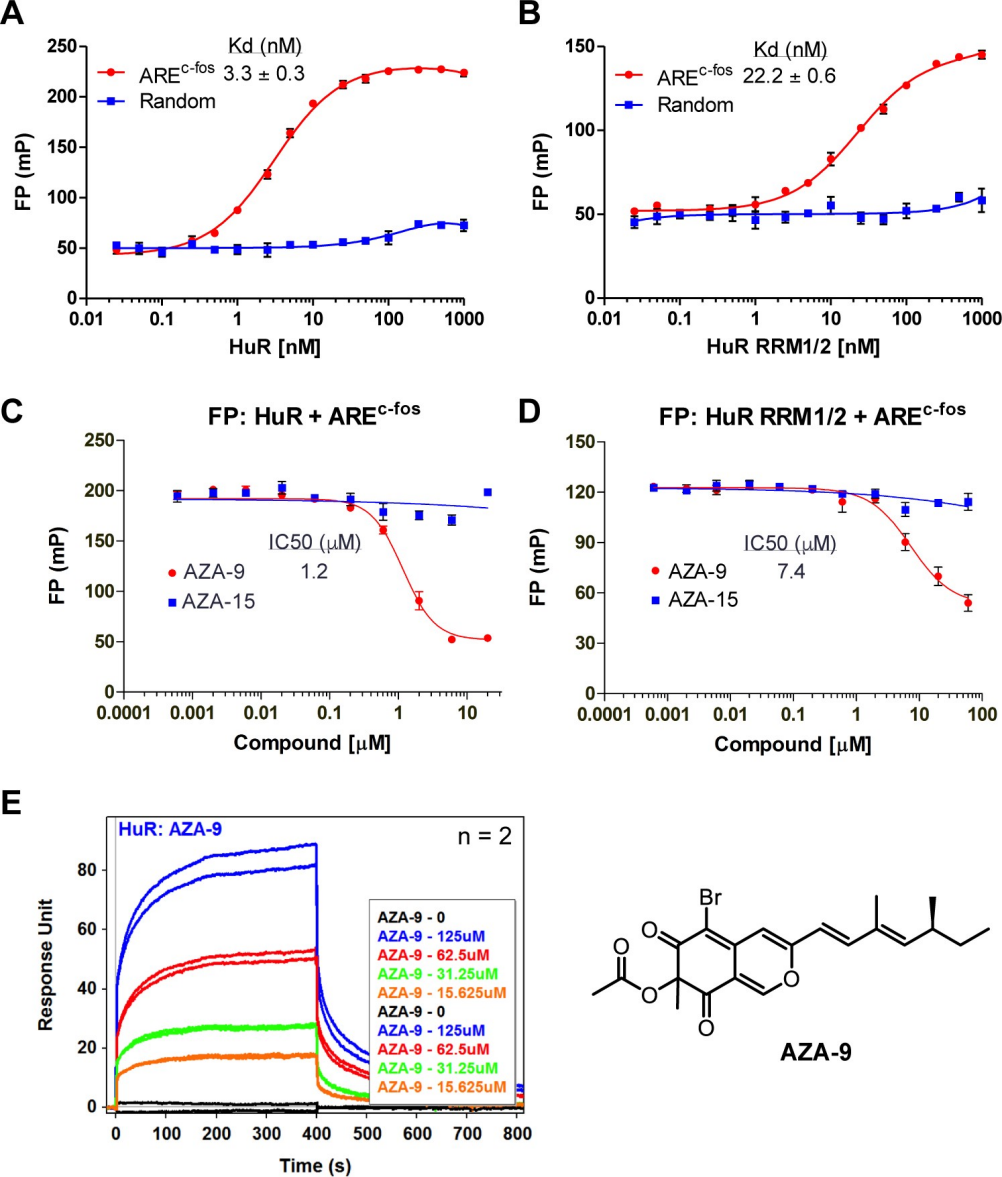
AZA-9 inhibits HuR-ARE RNA interaction and binds directly to HuR. Titration of 2 nM ARE^c-fos^ and control RNA (a 16-nt fluorescein-labeled RNA oligo with random sequence) with **(A)** HuR and **(B)** HuR RRM1/2 (n = 3). **(C)** Dose-response curve of AZA-9 disrupting HuR-ARE^c-fos^ binding in FP assay using 10 nM HuR and 2 nM fluorescein-labeled ARE^c-fos^ (n = 3). **(D)** Dose-response curve of AZA-9 disrupting HuR RRM1/2-ARE^c-fos^ binding in FP assay using 50 nM HuR RRM1/2 and 2 nM fluorescein-labeled ARE^c-fos^ (n = 3). **(E)** SPR sensorgrams of AZA-9 injected at increasing concentrations of 0–125 μM into a flow cell containing immobilized HuR (n = 2). Structure of AZA-9 is shown on the right. The following were the negative controls used–random RNA in **(A)** and **(B);** and AZA-15 in **(C)** and **(D)**.

A FP-based high throughput screen on HuR and a 16-mer ARE^Msi1^ RNA oligomer from the 3’-UTR of Musashi1 (Msi1) mRNA previously identified novel small molecule inhibitors of HuR-ARE interaction [22]. This FP assay [22] was used here to screen ~2000 compounds to identify more potent inhibitors of HuR-ARE interaction. The library contained 1673 compounds from the National Cancer Institute (Diversity Set II, Natural Products Set, and Approved Oncology Drugs Set) plus 291 in-house compounds. The screening identified a class of fungal natural products, azaphilones, as possible hits (S2 Fig). Azaphilones exhibit diverse biological effects, including cytotoxic, anti-inflammatory, anti-proliferative, and anti-tumorigenic activities [26, 35, 36]. The possible hits were azaphilones, which were compounds derived semi-synthetically from a fungal natural product, asperbenzaldehyde [22]. Multi-dose effect was determined to verify the inhibition of HuR-ARE interaction. All azaphilone derivatives tested (AZA-7 through AZA-14) except the negative control AZA-15, showed dose-dependent inhibition of HuR-ARE^Msi1^ interaction (S2 Fig). AZA-9 with a IC_50_ value of 1.1 μM (*n* = 3) was the most potent compound among the nine azaphilone derivatives and thus was chosen for further characterization. To confirm that the inhibition of HuR-ARE interaction is not mRNA specific and to further characterize the interaction between AZA-9 and HuR by NMR methods, we used another HuR target ARE, ARE^c-fos^, which is identical to the c-fos RNA sequence used in the crystal structure of HuR-RNA complex [8]. Similar to ARE^Msi1^, ARE^c-fos^ shows tight binding to full length HuR and a smaller fragment, HuR RRM1/2, with a Kd of 3.3 nM and 22.2 nM, respectively (Fig 1A and 1B). The ARE^c-fos^ interaction to either full length HuR (Fig 1C) or HuR RRM1/2 (Fig 1D) was disrupted in a dose-dependent manner upon addition of AZA-9, with IC_50_ value of 1.2 μM for full length HuR and 7.4 μM for HuR RRM1/2. SPR confirmed the direct binding of AZA-9 to HuR. Upon injections of increasing concentrations of AZA-9 on immobilized HuR, SPR sensorgrams showed increased optical response in a dose-dependent manner (Fig 1E). These findings indicate that AZA-9 disrupts HuR-ARE interaction through direct binding to HuR.

### NMR titrations of ^15^N-labeled HuR RRM1/2 with ARE^c-fos^

**Fig 2:**
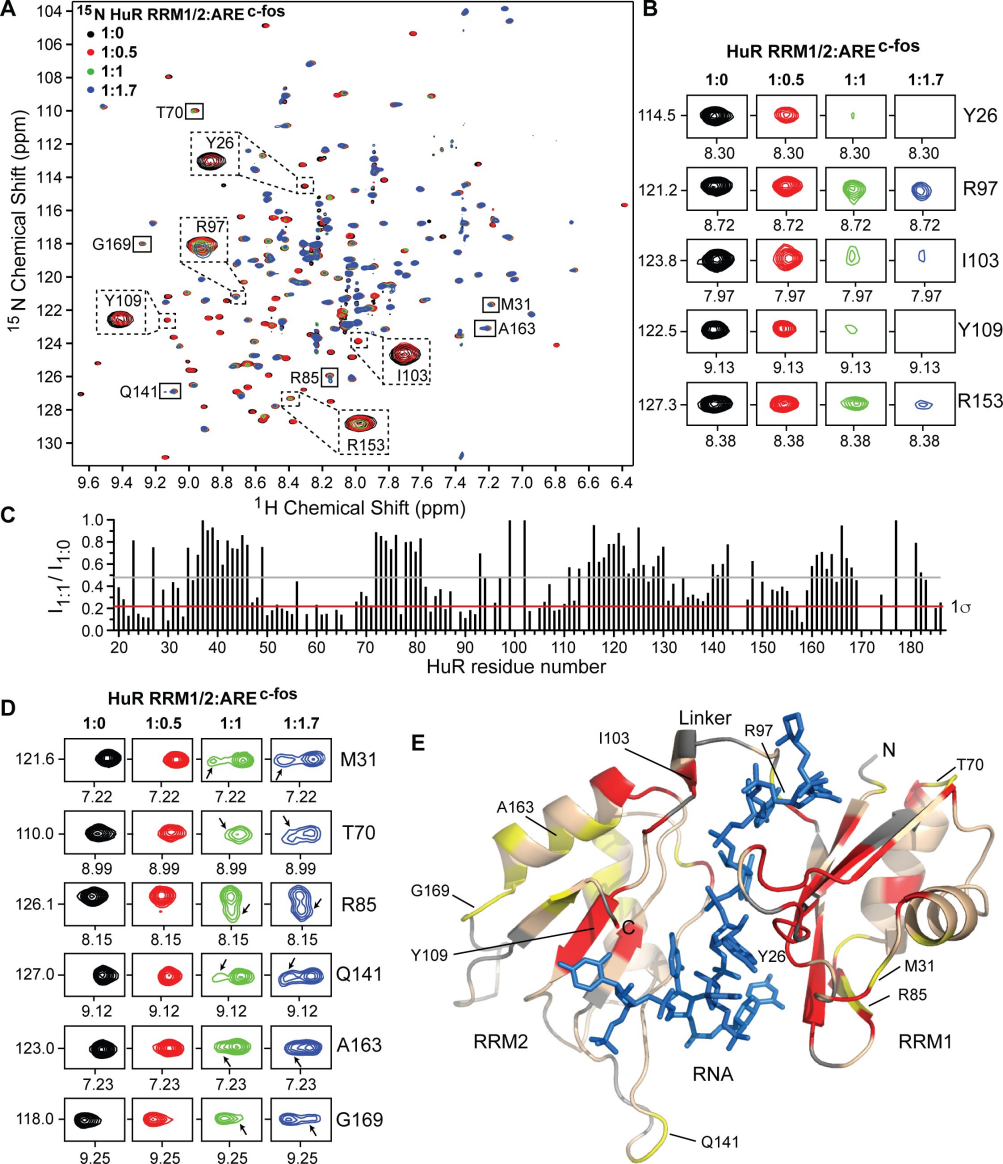
NMR titrations of ^15^N-labeled HuR RRM1/2 with ARE^c-fos^. **(A)** Overlay of four 2D ^1^H-^15^N TROSY spectra of ^15^N HuR RRM1/2 titrated with increasing molar ratios of ARE^c-fos^ RNA. Representative residues showing peak broadening (dashed box) and residues displaying changes in peak positions (solid box) are shown. **(B)** Representative residues displaying peak broadening upon RNA binding are shown at similar contour level at individual titration points. **(C)** Plot of relative peak intensity for all non-overlapping HuR RRM1/2 resonances in the ligand bound versus free state (I_1:1_ /I_1:0_). Gray and red lines correspond to the mean and one standard deviation from the mean (1σ), respectively. **(D)** Representative residues that displayed changes in peak positions are shown at similar contour level at individual titration points. While the original peak (RNA-free form) gradually decreases in intensity, a new peak (shown by arrow) appears and progressively gains intensity with increasing concentrations of RNA. **(E)** Results of NMR titrations mapped onto the co-crystal structure of HuR-ARE^**c-fos**^ complex (PDB 4ED5) and colored as follows: RRM1/2 residues with peak intensity ratio (I_1:1_ /I_1:0_) lower than 1σ (red), residues with new peaks shown in **D** (yellow), unassigned residues (gray), and RNA (blue).

Full length HuR gave non-ideal NMR spectra that made further NMR characterization challenging [20]. We therefore used a smaller fragment of HuR, HuR RRM1/2, which contains the essential domain needed for direct binding to ARE^c-fos^. The 2D ^1^H-^15^N TROSY spectrum of HuR RRM1/2 (Fig 2A) closely resembled the reported 2D ^1^H-^15^N HSQC spectrum, [20] thereby allowing use of the reported backbone amide assignments of HuR RRM1/2 [20] in our analysis. Overall, the 2D ^1^H-^15^N spectrum of HuR RRM1/2 is well dispersed with ~175 sharp, well-resolved peaks as expected for the construct (Fig 2A). To gain insights into the interaction of HuR RRM1/2 and RNA in solution, we used the same 11-mer c-fos RNA oligo (ARE^c-fos^) used in the co-crystallization of HuR RRM1/2 [8] for the NMR characterization of protein-RNA interaction. ^15^N-labeled HuR RRM1/2 was titrated with increasing concentrations of unlabeled ARE^c-fos^ at 1:0.5, 1:1, and 1:1.7 molar ratios, and the titration was monitored by acquiring 2D ^1^H-^15^N TROSY spectra. The stepwise addition of ARE^c-fos^ induced mainly peak broadening of specific HuR RRM1/2 resonances (Fig 2A) indicating that the interaction occurred in intermediate exchange NMR time scale for the backbone amides. Protein-RNA contacts have been identified in the co-crystal structure of HuR RRM1/2-ARE^c-fos^ [8]. Some essential HuR residues identified contributing to the specific recognition of RNA substrate through side-chain, main-chain and/or stacking interactions include residues Y26, R97, I103, Y109, and R153 [8]. These residues showed significant peak intensity reduction during the NMR titration (Fig 2B). To identify the amino acids involved in ARE^c-fos^ binding, we calculated the peak intensity ratio (I_1:1_/I_1:0_) for each non-overlapped peak at an HuR RRM1/2:RNA molar ratio of 1:1 (Fig 2C). Residues with peak intensity ratio lower than average intensity minus one standard deviation were mapped onto the co-crystal structure of the protein-RNA complex (Fig 2E). HuR RRM1/2 residues with significant peak intensity reduction cluster together in the β-strands, surrounding loops and linker region of RRM1/2 and form the RNA-binding surface of RRM1/2 (Fig 2E). The RNA interaction surface determined by NMR is consistent with the crystal structure of HuR-RNA complex.

Although the HuR RRM1/2 residues directly in contact with or in close proximity to the RNA showed reduction in peak intensities as described above, several residues distant from the RNA-binding site showed changes in peak positions in the presence of RNA (Fig 2D). Inspection of these peaks at lower contour level revealed the appearance of a new peak at a slightly different frequency with increasing concentrations of RNA. The original peak from the RNA-free form of HuR RRM1/2 gradually broadens, while the new peak emerging from the RNA-bound form progressively gains intensity with increasing amounts of RNA. HuR RRM1/2 residues displaying such shifts were mapped onto the structure of RNA-bound HuR (Fig 2E). Because these residues are situated ~9–13 Å apart from the RNA-binding site, such perturbations can be most directly interpreted a result of conformational change upon RNA binding. The appearance of two peaks corresponding to HuR RRM1/2 residues distant from the RNA-binding site in the presence of RNA is suggestive of slow conformational switching of HuR upon RNA binding. The allosteric effects observed in solution are in agreement with crystal structures and small-angle X-ray scattering (SAXS) analysis of RNA-free *versus* bound forms of HuR that demonstrate subtle conformational changes play a major role in formation of a stable compact HuR RRM1/2-RNA complex [8, 37].

### NMR assignment of ILV-labeled HuR RRM1/2

**Fig 3:**
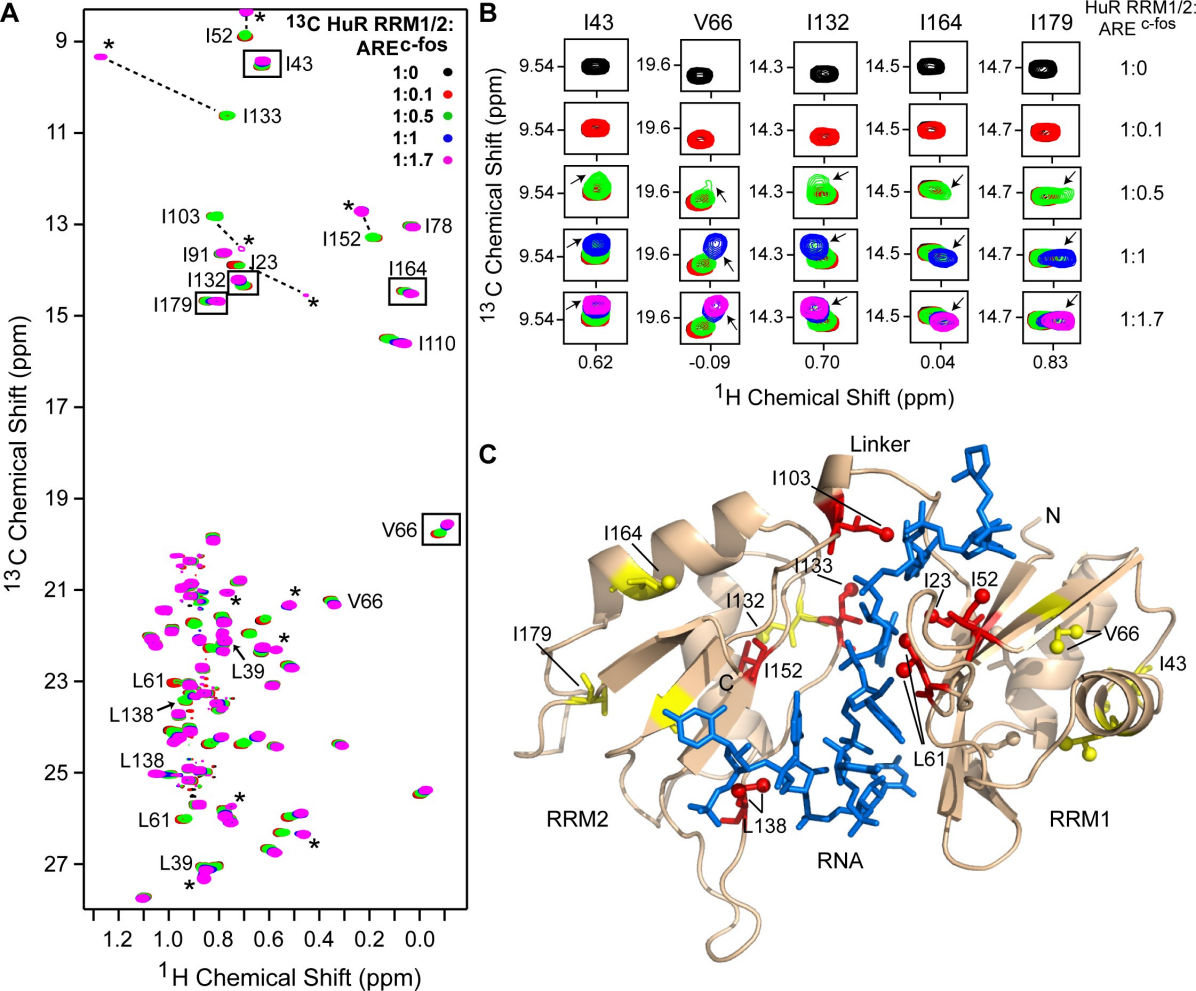
NMR titrations of ILV-labeled HuR RRM1/2 with ARE^c-fos^. **(A)** Overlay of five 2D ^1^H-^13^C HSQC spectra of ILV-labeled HuR RRM1/2 titrated with increasing concentrations of ARE^c-fos^. Assigned ILV peaks, the new peaks appearing upon complex formation (*), and some representative ILV residues away from the RNA-binding site that showed chemical shift perturbations (boxed) are indicated. **(B)** The boxed peaks in **A** are shown at a similar contour level at individual titration points. The new peak emerging with increasing concentrations of RNA is shown by an *arrow*. **(C)** Results of NMR titration mapped onto the co-crystal structure of HuR-ARE^**c-fos**^ complex (PDB 4ED5). Protein and RNA are represented as in Fig 2. Assigned ILV residues are shown as sticks and colored as follows: RRM1/2 residues showing new complex peaks (red) and residues with chemical shift perturbations as indicated in **B** (yellow).

ILV labeling offer additional probes in NMR studies because of their high sensitivity. Similar to the amide signals shown above, perturbations of ^13^C methyl peaks can be used to report on protein ligand interactions, conformational changes, structure and dynamics [28, 38, 39]. Further, side-chain interactions are crucial for the RNA recognition of HuR and methyl-containing residues such as isoleucine, leucine, and valine (ILV) occur near the RNA cleft. We therefore used ILV-labeled HuR RRM1/2 here to probe the side-chain protein-RNA interaction. His_6_-tagged HuR RRM1/2 contains 12 isoleucine, 15 leucine and 13 valine residues (the His-tag contributes 1 valine and 2 leucines). The 2D ^1^H-^13^C methyl HSQC spectrum of HuR RRM1/2 (Fig 3A) showed 12 Ile peaks (corresponding to each of the δ1 methyl group of 12 isoleucine) within the spectral window ~8–16 ^13^C ppm and 28 pairs of Leu and Val peaks (corresponding to the two δ1 and δ2 methyl groups of 15 leucines; and the two γ methyl groups of 13 valines) within 19–28 ^13^C ppm range. To assign the 16 ILV residues used herein, fifteen point mutations were introduced in HuR RRM1/2 (with isoleucine mutated to leucine; and leucine or valine mutated into isoleucine), and the resultant protein expressed and purified under native conditions. Comparable to the wild type construct, the mutant proteins were soluble at ~6 mg/mL in 100 mM NaCl, 10 mM sodium phosphate pH 6.8 buffer. To illustrate the assignment method to assign I23 for example, comparison of the 2D ^1^H-^13^C HSQC spectrum of the I23L point mutant that was selectively ^13^C-labeled at the isoleucine δ1 methyl with the spectrum of the wild type protein (S3A Fig), enabled the unambiguous assignment of the ^13^Cδ1 methyl resonance of I23. All the 12 isoleucine residues were assigned in addition to 3 leucines (L39, L61, and L138) and a valine residue, V66 (Fig 3A). These 16 ILV probes are strategically located and provide overall coverage of the HuR RRM1/2 structure.

### Titrations of ILV-labeled HuR RRM1/2 with ARE^c-fos^

To monitor the effect of RNA binding on ILV methyl resonances of HuR RRM1/2, 2D ^1^H-^13^C HSQC spectra were acquired on various titration samples. Of the assigned side chain methyl resonances, peaks corresponding to I23, I52, and L61 of RRM1, I103 of inter-domain linker region, and I133, L138, and I152 of RRM2 disappeared from their free position and reappeared at a different frequency in the spectrum as new peaks for the protein-RNA complex (Fig 3A). This indicates that the side-chains of these residues either directly mediate tight RNA-binding or are present in close proximity to the RNA and thus, experience strong perturbation in their local chemical environment upon RNA-binding. The affected ILV residues (I23, I52, L61, I103, I133, L138, and I152) delineate the RNA-binding cavity of HuR RRM1/2 and lie within 3–4 Å of the RNA substrate (Fig 3C).

Analogous to the allosteric effects observed in the backbone amide titrations (Fig 2D), several residues, such as L39, I43, V66 of RRM1, and I110, I132, I164, I179 of RRM2, with the side chain methyl group that are positioned ~12–18 Å from the RNA binding site also showed changes in their chemical shift positions (Fig 3B). The original free peak disappeared and a new peak representing the RNA-bound form of HuR appeared at a slightly different position (Fig 3B). After 1:1 complex is reached, it was observed that the addition of RNA merely adds to the intensity of the peak of the bound form. Residues with such perturbations are highlighted as *yellow* sticks in Fig 3C. The appearance of new peaks for such distant residues in the RNA-bound form confirms conformational changes upon RNA-binding. The results of side-chain ILV methyl titrations complement backbone amide titrations and together, they support that the ß-sheet region of HuR RRM1/2 is the RNA binding surface and RNA binding is accompanied by conformational changes in HuR.

### NMR titrations of HuR RRM1/2 with AZA-9

**Fig 4:**
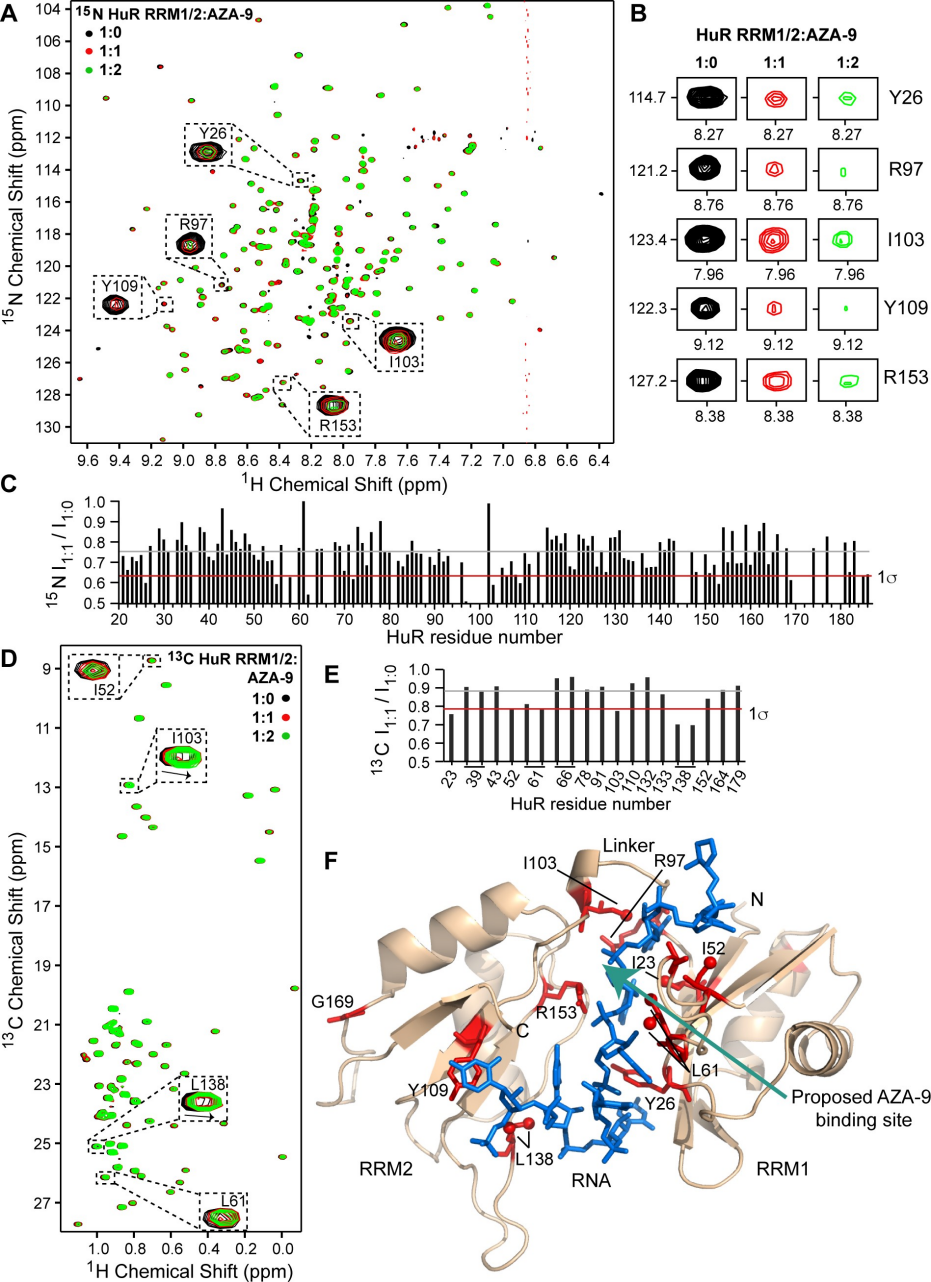
NMR titrations of HuR RRM1/2 with AZA-9. **(A)** Overlay of three 2D ^1^H-^15^N TROSY spectra of ^15^N HuR RRM1/2 titrated with increasing molar ratios of AZA-9. The peaks of some critical RNA-binding residues that undergo significant line broadening upon addition of AZA-9 are shown (expanded dashed box). **(B)** Representative residues showing peak broadening upon titration of AZA-9 are shown at similar contour level at individual titration points. **(C)** Relative peak intensity plot for non-overlapping amide resonances of HuR RRM1/2 in the ligand bound versus free state (I_1:1_ /I_1:0_). **(D)** Overlay of three 2D ^1^H-^13^C HSQC spectra of ILV-labeled HuR RRM1/2 titrated with increasing molar ratios of AZA-9. Analogous to ^15^N-titrations, ILV methyl groups of RNA-binding residues showed peak broadening with a few residues such as I103 and L138 also displaying chemical shift deviations (dashed box). **(E)** Relative peak intensity plot for assigned ILV methyl HuR RRM1/2 resonances in the ligand bound versus free state (I_1:1_ /I_1:0_). **(F)** Results of titrations mapped onto the co-crystal structure of HuR-ARE^**c-fos**^ complex (PDB 4ED5). Protein and RNA are shown as in Figs 2 and 3. RRM1/2 residues with peak intensity ratio (I_1:1_ /I_1:0_) lower than 1σ are colored red and the proposed AZA-9 binding site is shown by an *arrow*. Most of the AZA-9 affected residues are key RNA-binding residues. **(C,E)** Gray and red lines correspond to the mean and one standard deviation from the mean (1σ), respectively.

The interaction of AZA-9 with HuR RRM1/2 was characterized by NMR methods. Titrations of ^15^N/ILV-labeled HuR RRM1/2 with AZA-9 resulted in concentration dependent reduction in the ^15^N and ILV peak intensities of HuR RRM1/2 (Fig 4), indicating complex formation on an intermediate exchange time scale. In addition to the decrease in peak intensities, the side chain methyl groups of some HuR RRM1/2 residues, such as I103, L138 (Fig 4D), also showed chemical shift deviations upon binding of AZA-9. Further, key RNA-binding residues, Y26, R97, I103, Y109, and R153 (Fig 2B), exhibited significant peak broadening with increasing doses of AZA-9 in the ^15^N-titrations (Fig 4B). Specifically, residue R97 in the inter-domain linker region (whose intensity reduced by ~50% at equimolar concentration of AZA-9, Fig 4C) has been previously reported as important for RNA binding, RNA recognition and high affinity HuR-RNA complex formation [8]. Comparable to the results of the amide titrations, results of the ILV titrations of other RNA-binding residues, such as I52, L61, I103, and L138, identified earlier (Fig 3A) also displayed significant reduction in peak intensities upon addition of AZA-9 (Fig 4D). A plot of the peak intensity ratio (I_1:1_/I_1:0_) in the bound and free form at 1:1 molar ratio (Fig 4) revealed that the NMR resonances of the major RNA-binding residues of HuR, including K55, G62, R97, I103, L138, and R153, were significantly perturbed by AZA-9. Residues that were significantly perturbed during titrations were mapped on the structure of RNA-bound HuR (Fig 4F). Complex formation with AZA-9 primarily affected a cluster of RNA-binding residues located near the inter-domain linker region of HuR (Fig 4F). NMR results indicate that AZA-9 interacts at the same binding pocket that HuR RRM1/2 uses to bind its target RNA.

### *In silico* docking of AZA-9 in the RNA cleft of HuR RRM1/2

**Fig 5:**
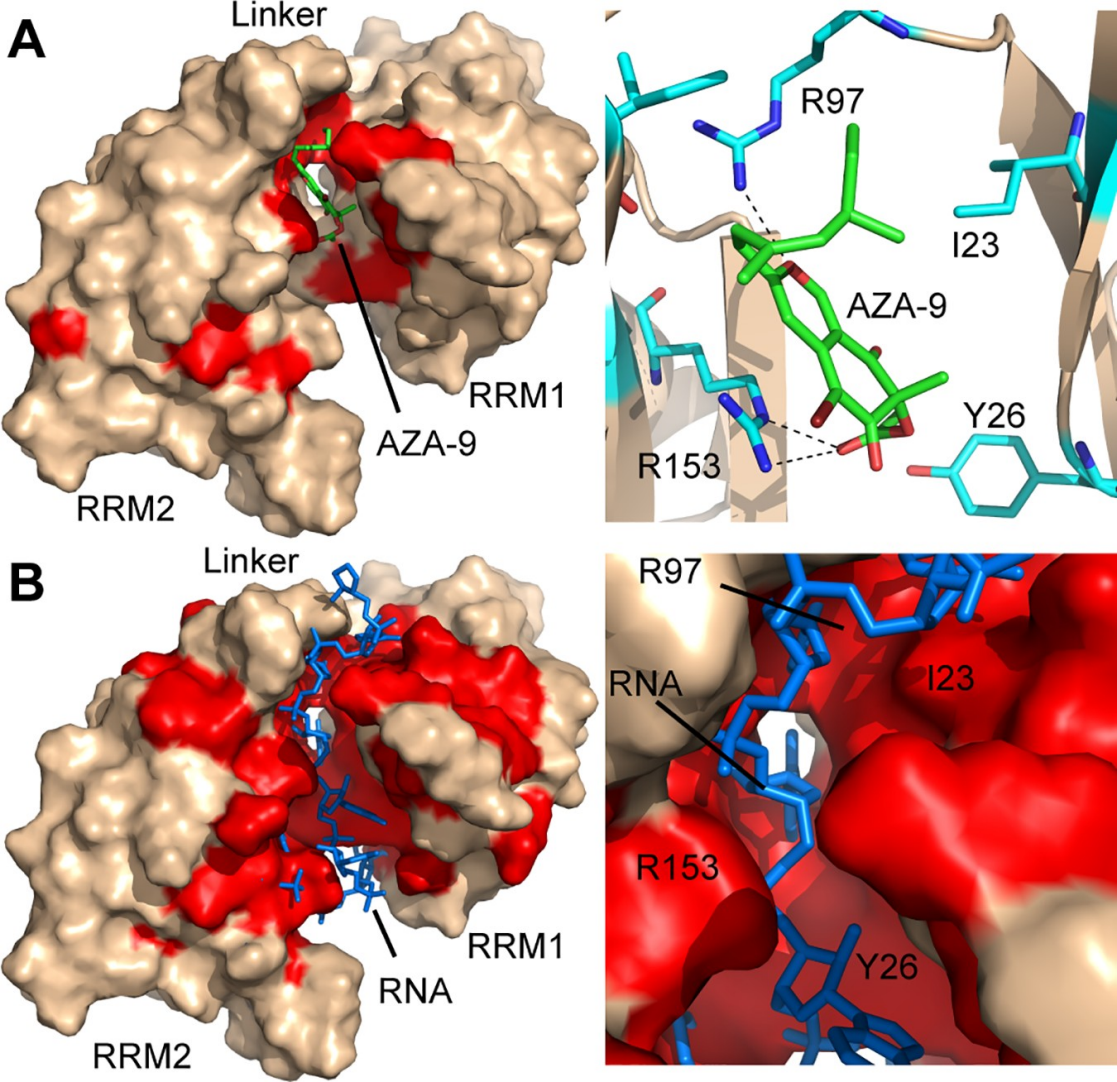
Molecular docking identifies a possible binding pocket for AZA-9 in the RNA-cleft of HuR. **(A)** Computational model of AZA-9 bound to HuR RRM1/2, with protein shown in surface representation and AZA-9 in sticks. HuR RRM1/2 residues affected in AZA-9 NMR titrations are colored red. An expanded view of the binding pocket is shown in the right. Residues involved in AZA-9 binding are displayed as sticks, with hydrogen bonds shown as dashed lines. **(B)** Surface representation of HuR RRM1/2 bound to RNA (sticks) for comparison with the AZA-9 model. Residues affected upon RNA titrations are highlighted in red. An expanded view of the RNA-pocket is shown in the right. Residues (Y26, K55, L61, R97, R153) perturbed by AZA-9 in the NMR titrations are critical RNA-binding residues.

Molecular docking studies were performed to gain further insight into the binding mode of compound AZA-9 to HuR. Fig 5A represents the top-scoring computational model of compound AZA-9 bound HuR RRM1/2 generated using FRED [31], followed by full-atom minimization with ROSETTA [33]. The resulting model had a score of -304.85 Rosetta Energy Units and an interface score (the difference between the score of the complex and the sum of the scores of the protein and AZA-9 alone) of -14.94 Rosetta Energy Units. Several hydrophobic and positively charged HuR residues, such as Y26, K55, R97, and R153 line the pocket for AZA-9 and potentially stabilize the protein-ligand complex through electrostatic, hydrogen bond, hydrophobic, and pi-stacking interactions (Fig 5A). Consistent with the NMR-derived binding site of AZA-9 (Fig 4F), molecular docking confirmed a possible binding mode for compound AZA-9 in the RNA binding cleft of HuR near the inter-domain linker region (Fig 5B). Together, results of NMR titrations and molecular docking indicate that compound AZA-9 disrupts HuR-RNA interaction by competitively binding in the RNA cleft of HuR (Fig 5).

## Discussion

HuR-ARE interaction [1, 4–6] contributes to carcinogenesis by stabilizing the mRNAs of oncogenes [7, 12, 15, 18, 19], thus, finding inhibitors of HuR-ARE interaction could contribute to the development of new cancer therapies [20, 22]. So far, there are a limited number of HuR inhibitors [16, 20–25] that competitively bind to HuR and directly disrupt HuR-ARE interactions [20–22]. Currently, the most potent HuR inhibitor known, MS-444, is a bacterial natural product isolated from *Actinomyces* sp. microbial broths, and MS-444 inhibits HuR-RNA interaction by interfering with HuR homodimerization [21]. Here, we identified a new class of compounds, azaphilones (S2 Fig) [35, 36] and in particular, AZA-9 (Fig 1), as novel inhibitors of HuR-ARE interaction.

The azaphilones studied here are derived from the fungal secondary metabolite asperbenzaldehyde [26, 27]. Fungi-derived natural products are excellent sources of pharmaceuticals and many fungal secondary metabolites show anti-cancer properties that inhibit cell proliferation, angiogenesis, and tumorigenesis.[35, 40, 41] The two rings of azaphilones form the isochromene scaffold, and this scaffold is present in the previously identified methyl-benzoisochromene scaffold of the chrysanthones secondary fungal metabolites isolated from another fungus, *Ascochyta chrysanthemi*.[41] Chrysanthones were reported to have anti-proliferative, anti-tumorigenic and anti-angiogenic properties, however, their specific molecular target was not determined.[41] The presence of the isochromene scaffold plus the observed anti-cancer properties of chrysanthones could be associated to HuR inhibition.

The tandem RRM1/2 of HuR is the minimal domain needed for binding AREs. Our results of the backbone amide titrations showed significant peak broadening for the ARE-binding residues similar to what was reported by Wang et al. [20] (Fig 2), however, we also observed additional new slow exchange peaks in the ILV titrations (Fig 3). This differing NMR exchange behavior could be due to the direct interactions of the protein side chains with the RNA substrate and their dominant role in the formation of tight HuR RRM1/2-ARE complex [8]. Additionally, we observed allosteric effects in HuR RRM1/2 upon RNA binding (Figs 2D and 3B). Conformational changes in HuR RRM1/2 on binding the RNA substrate have been previously reported [8, 37]. These conformational changes contribute in the formation of a stable, high-affinity HuR RRM1/2-RNA complex. Consistent with the crystal structures and SAXS analysis [8, 37], our NMR results identified the specific residues (such as M31, T70, R85, Q141, A163, and G169) that are involved in the slow conformational switching in HuR RRM1/2 upon RNA binding (Figs 2 and 3).

The crystal structures of the free and RNA-bound HuR RRM1/2 [8] suggest major structural rearrangements in the relative orientation of RRM1 with respect to RRM2 upon RNA binding (S4 Fig). For example, RRM2 has to swing about 41 Å to reposition itself vis-à-vis RRM1 upon RNA binding. This major conformational change in HuR RRM1/2 upon RNA binding is not reflected in the results of our NMR titrations as well as the results of the amide titrations of Wang et al. [20]. For such major conformational rearrangements of the two RRM domains, one would expect major changes in the peak positions in the ^15^N TROSY spectra of the free and RNA-bound HuR RRM1/2 (Fig 2). Instead, we observed essentially similar peak positions in the ^15^N TROSY of free and RNA-bound HuR RRM1/2 (Fig 2), with the peak intensities of the RNA-bound form progressively weakening upon addition of more RNA. In the ILV-titrations (Fig 3), there were indeed new slow-exchange peaks for RRM2 isoleucine residues (I103, I133, I152) but the rest of the isoleucines in RRM2 (I110, I164, and I179) were essentially in similar (fast exchange) peak positions as the free form suggesting the changes in the peak positions and intensities observed by NMR are due to RNA-binding rather than the major conformational rearrangements of the two domains upon RNA-binding. Our NMR results suggest that RRM1 and RRM2 are somewhat ‘pre-formed’ for RNA-binding with the two domains already close together and poised to accept the RNA. Upon RNA-binding, the side chains and loops of HuR RRM1/2 ‘wiggle’ to accommodate and interact with the RNA. SAXS results suggest two populations of free HuR RRM1/2 where one population has an extended structure (with a size of 74 Å) and another population that has a more compact structure (with a size of 56 Å) [37]. Upon RNA binding, the HuR RRM1/2 becomes even more compact (with a size of 51 Å). This suggest that SAXS is able to trap two populations of free HuR RRM1/2 whereas our NMR results suggest an average conformation.

Our efforts to co-crystallize AZA-9 with HuR RRM1/2 have been so far unsuccessful, thus, we used NMR methods to characterize how AZA-9 interacts with HuR RRM1/2. Results of NMR titrations showed that AZA-9 essentially perturbs the same HuR RRM1/2 residues involved in binding RNA (Fig 4). NMR titrations with AZA-9 affected specific RNA-binding residues of HuR RRM1/2 (Fig 4). The previously identified key ARE-binding residues, including Y26, L61, R97, I103, Y109, and R153 that make side chain, main chain and/or stacking interactions with the ARE substrate [8] showed significant NMR perturbations upon the addition of AZA-9 (Fig 4A and 4D). In particular, R97, in the inter-domain linker region, which showed the strongest peak intensity reduction has been identified through mutagenesis as a critical residue required for high affinity ARE binding [8]. Results of both backbone amide and ILV titrations indicate that AZA-9 predominantly affect a surface near the inter-domain linker region in the RNA cleft of HuR (Fig 4F).

In agreement with the NMR-derived binding site of AZA-9, results of molecular docking positioned AZA-9 in the ARE-binding cleft near the inter-domain linker region of HuR RRM1/2 (Fig 5A). Computational modeling suggested that AZA-9 is well situated in the binding pocket surrounded by several positively charged and hydrophobic residues to enable hydrophobic, hydrogen bond, and/or electrostatic interactions. In the computational model, the long hydrophobic tail of AZA-9 lies adjacent to the methyl side chain of I23, and runs roughly parallel to the aliphatic chain of R97 to promote hydrophobic interactions; the oxygen atom of the pyran ring in AZA-9 positioned such it forms a hydrogen bond with the guanidinium group of R97; and the ester carbonyl of AZA-9 is positioned very close to the guanidinium side chain of R153, forming a salt bridge. The interaction of AZA-9 with R97 is particularly notable as R97 was found by crystallography [8] to be a key residue for stable HuR-ARE complex formation. Overall, our results show that AZA-9 competes with target ARE for binding in the RNA cleft of HuR RRM1/2.

There are several HuR RRM1/2 residues (S88, S100, T118, S158, and K182) that are involved in the phosphorylation and ubiquitinylation of HuR, and these posttranslational modifications affect the RNA-binding, nucleo-cytoplasmic shuttling, and protein stability of HuR ([11]). Of these residues, S100 is closest in distance, within 5Å, to the predicted AZA-9 binding site, and phosphorylation of S100 could be affected by AZA-9 binding. The other residues (S88, T118, S158, and K182) are too far away from the proposed AZA-9 binding site, and are not expected to make direct contact with AZA-9. The effect on the posttranslational sites should be explored for the second-generation inhibitors designed on the AZA-9 scaffold that show *in vivo* potency.

Our results indicate that the binding of AZA-9 to HuR is weak, occurring at about micromolar range (Fig 1). This weak binding suggests that AZA-9 may not be specific to HuR, and indeed, our unpublished data indicate that AZA-9 also binds to another RRM-containing protein, Musashi-1 (Msi1). Musashi-1 contains two RRMs, which share on average about 26% sequence identity and 41% sequence similarity with HuR RRM1/2. Nevertheless, our NMR and modeling results (Figs 4 and 5) showing how AZA-9 binds to the RNA-binding pocket of HuR RRM1/2 point the direction in designing improved versions of AZA-9 that could yield tighter-binding compounds that are more specific to HuR, and yield more potent inhibitors of HuR, that can be used for developing new anticancer therapies.

To summarize, azaphilones, and in particular, AZA-9, which are derived from fungal natural products, bind to the HuR RNA-binding domain, RRM1/2. Binding of AZA-9 to HuR disrupted HuR-RNA interaction. SPR, NMR and molecular docking confirmed that AZA-9 inhibited HuR-RNA interaction by competing directly for the RNA-binding site in HuR.

## Supporting information

S1 Fig Protein gel of purified full length HuR and the HuR RRM1/2. Coomassie stained SDS-PAGE of purified recombinant full length HuR (*FL HuR*, 36 kDa) and the RRM1/2 fragment (24 kDa). Lanes were loaded with *low* and *high* amounts of purified proteins.
(TIF)

S2 Fig Azaphilones inhibit HuR-RNA interaction. **(A)** Structures of azaphilone compounds tested in the inhibition assay. **(B)** Dose-response curves of azaphilones disrupting HuR-ARE^Msi1^ binding in FP assay using 10 nM HuR and 2 nM fluorescein-labeled ARE^Msi1^ RNA. Data are representative of three independent experiments. AZA-15 does not inhibit HuR- ARE^Msi1^ interaction and serves as negative control; all other azaphilone derivatives show dose-dependent inhibition of HuR-ARE^Msi1^ interaction.
(TIF)

S3 Fig ILV assignments of HuR RRM1/2 by mutagenesis. 2D ^1^H-^13^C HSQC spectra for HuR RRM1/2 mutants (red) overlaid with the wild type spectra (black). **(A-K)** A single missing ^13^Cδ1 methyl peak for Ile; and **(L)** two ^13^Cδ1 and ^13^Cδ2 methyl peaks for Leu corresponding to the mutated residue are indicated.
(TIF)

S4 Fig Superposition of the RNA-bound and the RNA-free crystal structures of HuR RRM1/2 suggests a large (41 Å) interdomain swinging motion of RRM2 upon RNA-binding. Our NMR data does not detect this large interdomain motion of RRM1 and RRM2 upon RNA-binding. Crystal structures of **(A)** RNA-bound (PDB ID: 4ED5) and **(B)** RNA-free HuR RRM1/2 (PDB ID: 4EGL). **(C)** Superposition of the **(A)** RNA-bound and the **(B)** RNA-free forms of HuR RRM1/2. The structures are superimposed on the RRM1 domain. The structures are colored as follows: RRM1 (green), the inter-domain linker region (yellow), RRM2 (orange), and RNA (blue).
(TIF)
