# Supplementary figures and images for: The fungal natural product azaphilone-9 binds to HuR and inhibits HuR-RNA interaction in vitro
Source: PLoS One. 2017 Apr 17;12(4):e0175471. doi: 10.1371/journal.pone.0175471 (PMC5393604; doi:10.1371/journal.pone.0175471)

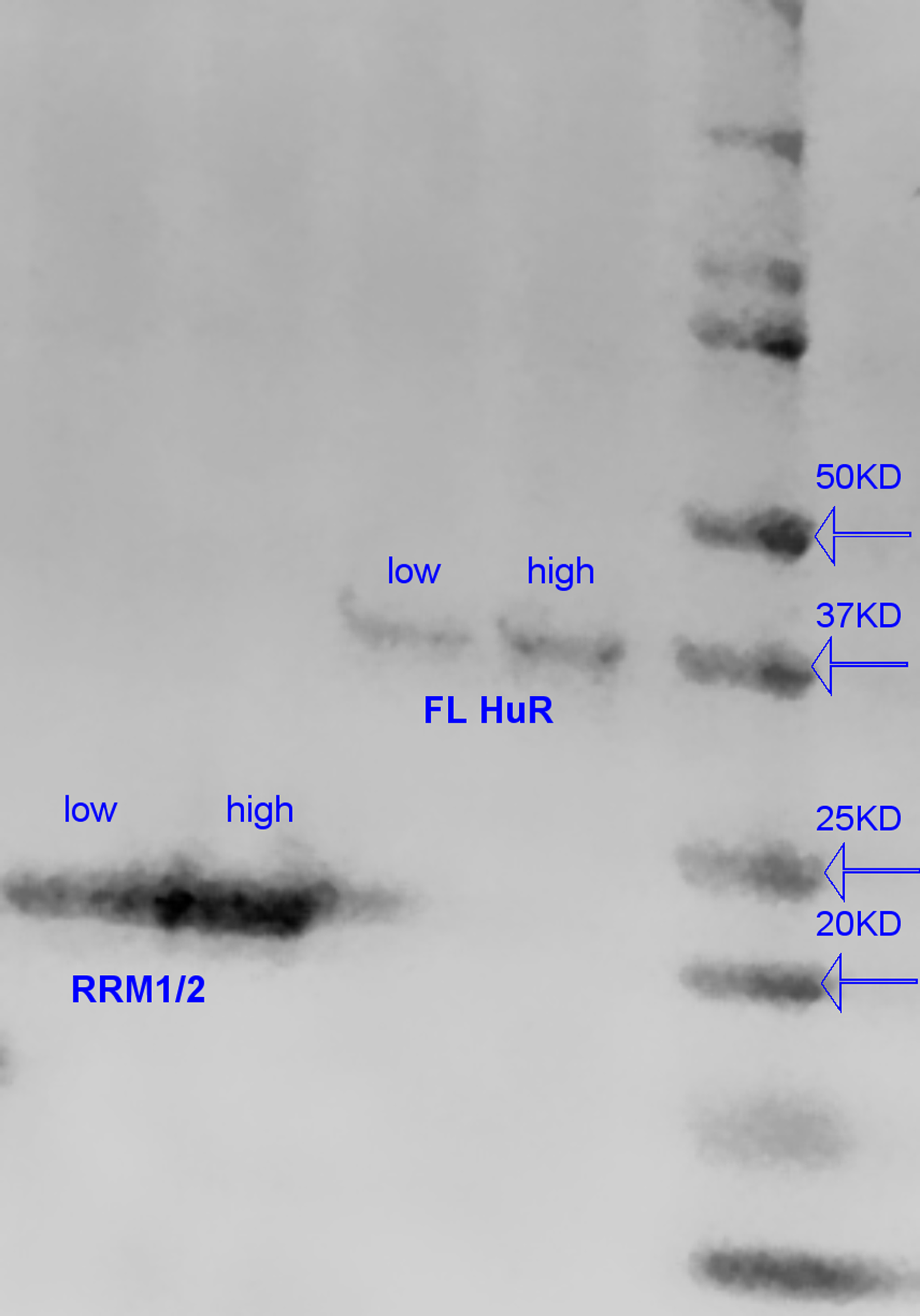

Supplement: S1 Fig — Coomassie stained SDS-PAGE of purified recombinant full length HuR (FL HuR, 36 kDa) and the RRM1/2 fragment (24 kDa). Lanes were loaded with low and high amounts of purified proteins. (TIF) [file pone.0175471.s001.tif]

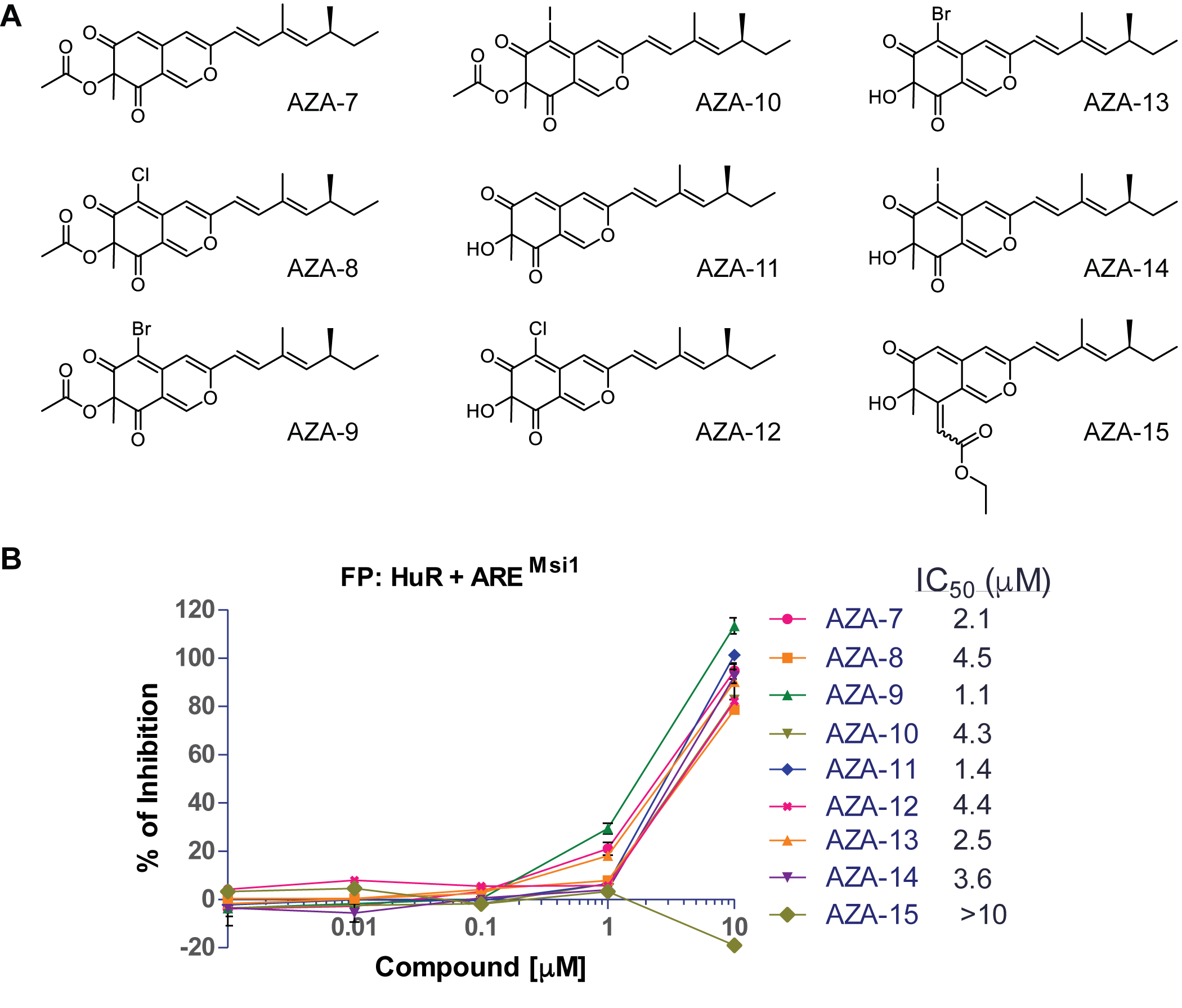

Supplement: S2 Fig — (A) Structures of azaphilone compounds tested in the inhibition assay. (B) Dose-response curves of azaphilones disrupting HuR-AREMsi1 binding in FP assay using 10 nM HuR and 2 nM fluorescein-labeled AREMsi1 RNA. Data are representative of three independent experiments. AZA-15 does not inhibit HuR- AREMsi1 interaction and serves as negative control; all other azaphilone derivatives show dose-dependent inhibition of HuR-AREMsi1 interaction. (TIF) [file pone.0175471.s002.tif]

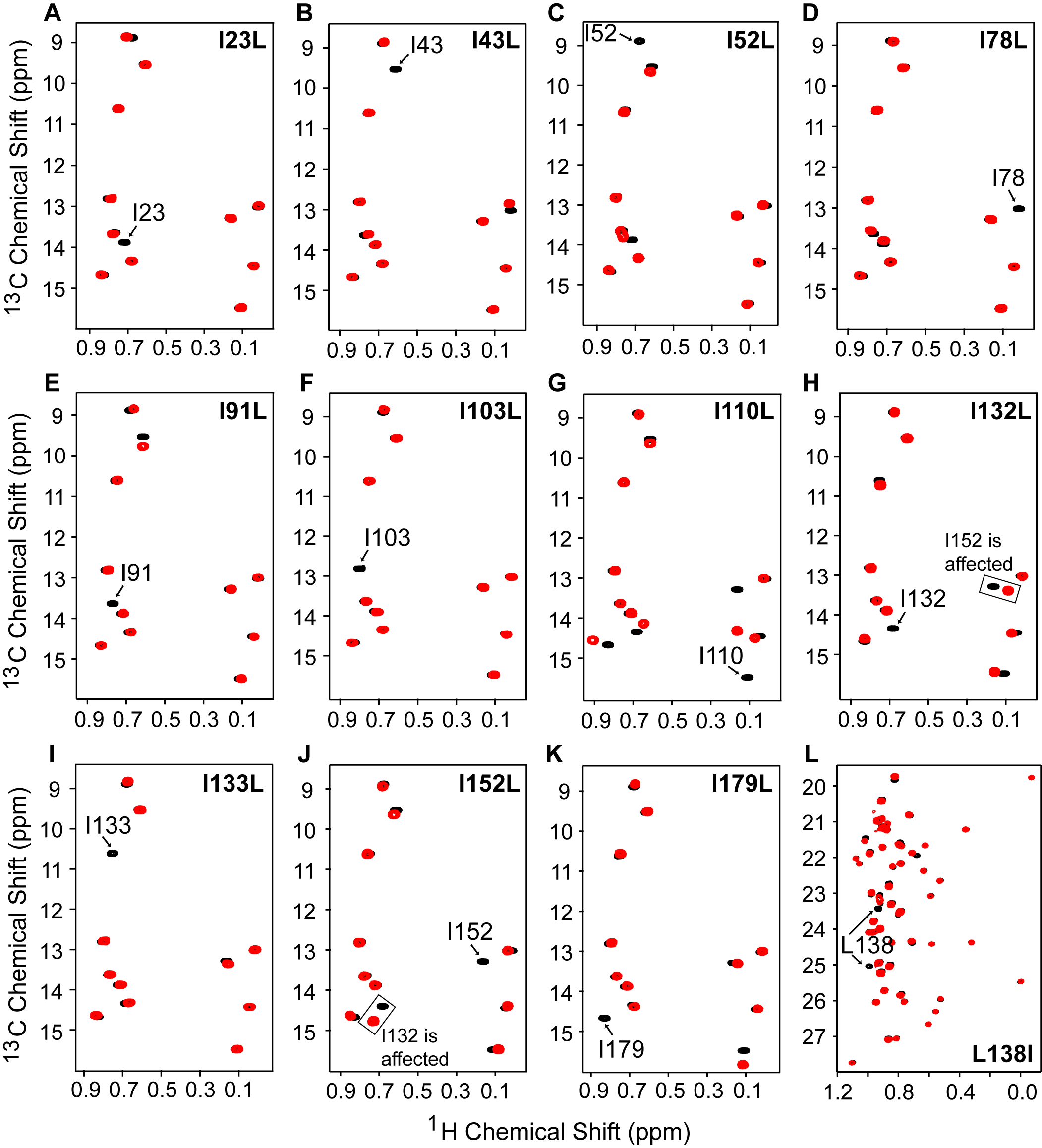

Supplement: S3 Fig — 2D 1H-13C HSQC spectra for HuR RRM1/2 mutants (red) overlaid with the wild type spectra (black). (A-K) A single missing 13Cδ1 methyl peak for Ile; and (L) two 13Cδ1 and 13Cδ2 methyl peaks for Leu corresponding to the mutated residue are indicated. (TIF) [file pone.0175471.s003.tif]

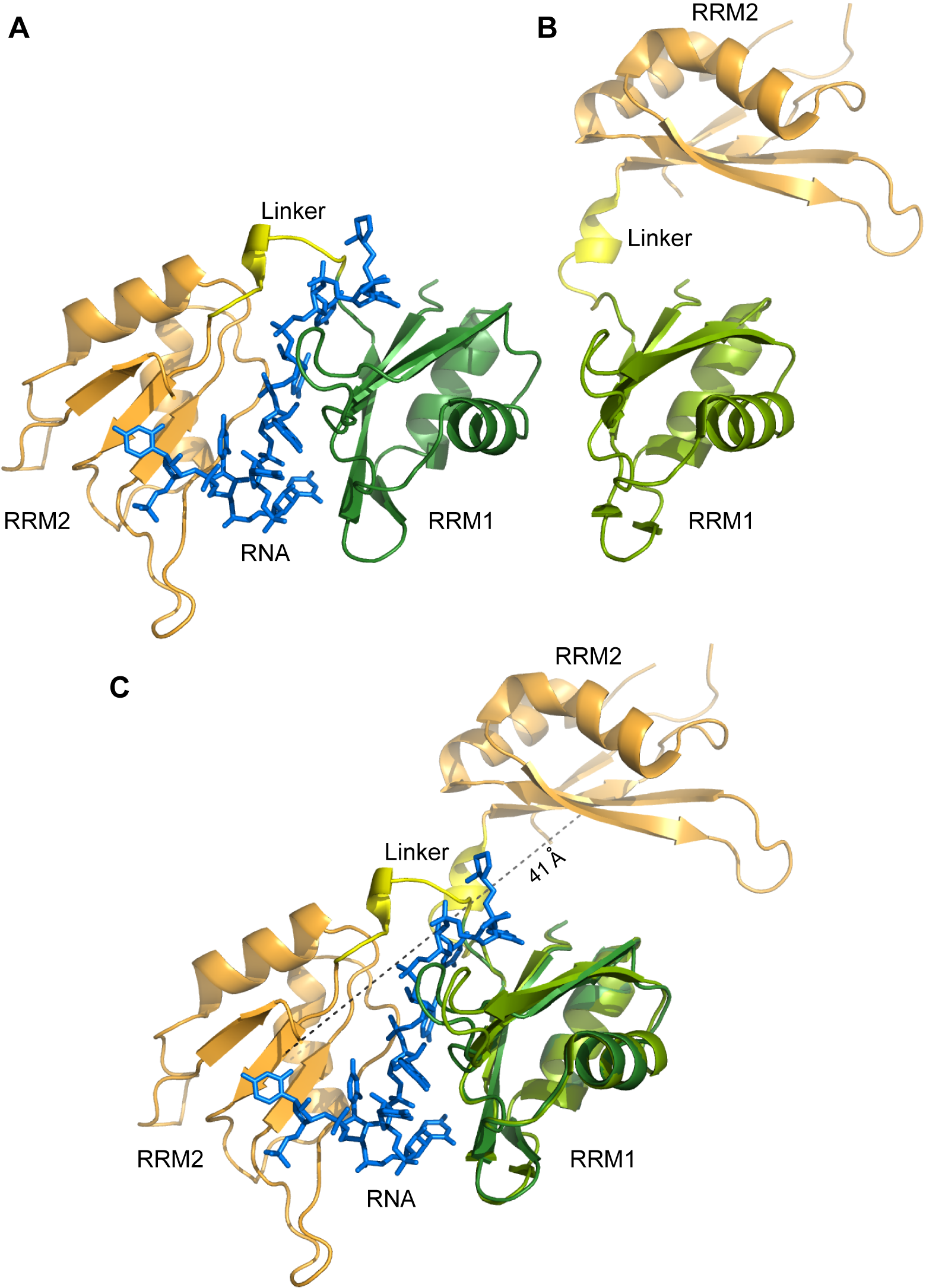

Supplement: S4 Fig — Our NMR data does not detect this large interdomain motion of RRM1 and RRM2 upon RNA-binding. Crystal structures of (A) RNA-bound (PDB ID: 4ED5) and (B) RNA-free HuR RRM1/2 (PDB ID: 4EGL). (C) Superposition of the (A) RNA-bound and the (B) RNA-free forms of HuR RRM1/2. The structures are superimposed on the RRM1 domain. The structures are colored as follows: RRM1 (green), the inter-domain linker region (yellow), RRM2 (orange), and RNA (blue). (TIF) [file pone.0175471.s004.tif]
